# Curva de Aprendizagem da Mortalidade Hospitalar da Substituição da Válvula Aórtica Transcateter: Insights do Registro Nacional Brasileiro

**DOI:** 10.36660/abc.20230622

**Published:** 2024-07-10

**Authors:** Fernando Luiz de Melo Bernardi, Alexandre A. Abizaid, Fábio Sândoli de Brito, Pedro A. Lemos, Dimytri Alexandre Alvim de Siqueira, Ricardo Alves Costa, Rogério Eduardo Gomes Sarmento Leite, Fernanda Marinho Mangione, Luiz Eduardo Koenig São Thiago, José A. Mangione, Valter Correia de Lima, Adriano Dourado Oliveira, Marcos Antônio Marino, Carlos José Francisco Cardoso, Paulo R. A. Caramori, Rogério Tumelero, Antenor Lages Fortes Portela, Mauricio Prudente, Leônidas Alvarenga Henriques, Fabio Solano Souza, Cristiano Guedes Bezerra, Guy F. A. Prado, Leandro Zacaris Figueiredo Freitas, Ederlon Ferreira Nogueira, George César Ximenes Meireles, Renato Bastos Pope, Enio Guerios, Pedro Beraldo de Andrade, Luciano de Moura Santos, Mauricio Felippi de Sá Marchi, Nelson Henrique Fantin Fundão, Henrique Barbosa Ribeiro

**Affiliations:** 1 Hospital das Clínicas Faculdade de Medicina Universidade de São Paulo São Paulo SP Brasil Instituto do Coração do Hospital das Clínicas da Faculdade de Medicina da Universidade de São Paulo, São Paulo, SP – Brasil; 2 Hospital Sírio-Libanês São Paulo SP Brasil Hospital Sírio-Libanês, São Paulo, SP – Brasil; 3 Hospital Israelita Albert Einstein São Paulo SP Brasil Hospital Israelita Albert Einstein, São Paulo, SP – Brasil; 4 Instituto Dante Pazzanese de Cardiologia São Paulo SP Brasil Instituto Dante Pazzanese de Cardiologia, São Paulo, SP – Brasil; 5 Instituto de Cardiologia Porto Alegre RS Brasil Instituto de Cardiologia, Porto Alegre, RS – Brasil; 6 Hospital Beneficência Portuguesa de São Paulo São Paulo SP Brasil Hospital Beneficência Portuguesa de São Paulo, São Paulo, SP – Brasil; 7 Hospital SOS Cárdio Florianópolis SC Brasil Hospital SOS Cárdio, Florianópolis, SC – Brasil; 8 Santa Casa de Misericórdia de Porto Alegre Porto Alegre RS Brasil Santa Casa de Misericórdia de Porto Alegre, Porto Alegre, RS – Brasil; 9 Hospital Santa Izabel Salvador BA Brasil Hospital Santa Izabel, Salvador, BA – Brasil; 10 Hospital Madre Teresa Belo Horizonte MG Brasil Hospital Madre Teresa, Belo Horizonte, MG – Brasil; 11 Hospital Naval Marcilio Dias Rio de Janeiro RJ Brasil Hospital Naval Marcilio Dias, Rio de Janeiro, RJ – Brasil; 12 Hospital São Lucas PUCRS Porto Alegre RS Brasil Hospital São Lucas da PUCRS, Porto Alegre, RS – Brasil; 13 Universidade de Passo Fundo Passo Fundo RS Brasil Universidade de Passo Fundo, Passo Fundo, RS – Brasil; 14 Associação Piauiense de Combate ao Câncer Teresina PI Brasil Associação Piauiense de Combate ao Câncer, Teresina, PI – Brasil; 15 Hospital Encore Goiânia GO Brasil Hospital Encore, Goiânia, GO – Brasil; 16 Hospital Albert Sabin Juiz de Fora MG Brasil Hospital Albert Sabin, Juiz de Fora, MG – Brasil; 17 Hospital Universitário Professor Edgard Santos Salvador BA Brasil Hospital Universitário Professor Edgard Santos, Salvador, BA – Brasil; 18 Hospital Cardio-Pulmonar Salvador BA Brasil Hospital Cardio-Pulmonar, Salvador, BA – Brasil; 19 Hospital Aliança Rede D´Or Salvador BA Brasil Hospital Aliança Rede D´Or, Salvador, BA – Brasil; 20 Universidade Federal de Goiás Goiânia GO Brasil Universidade Federal de Goiás, Goiânia, GO – Brasil; 21 Hospital do Coração de Londrina Londrina PR Brasil Hospital do Coração de Londrina, Londrina, PR – Brasil; 22 Instituto de Assistência Médica ao Servidor Público Estadual de São Paulo São Paulo SP Brasil Instituto de Assistência Médica ao Servidor Público Estadual de São Paulo, São Paulo, SP – Brasil; 23 Hospital Hans Dieter Schmidt Joinville SC Brasil Hospital Hans Dieter Schmidt, Joinville, SC – Brasil; 24 Universidade Federal do Paraná Hospital de Clinicas Curitiba PR Brasil Universidade Federal do Paraná - Hospital de Clinicas, Curitiba, PR – Brasil; 25 Santa Casa de Misericórdia de Marília Marilia SP Brasil Santa Casa de Misericórdia de Marília, Marilia, SP – Brasil; 26 Hospital Santa Lúcia Brasília DF Brasil Hospital Santa Lúcia, Brasília, DF – Brasil

**Keywords:** Substituição da Valva Aórtica Transcateter, Estenose da Valva Aórtica, Mortalidade Hospitalar

## Abstract

**Fundamento:**

Dados robustos sobre a curva de aprendizagem (LC) da substituição da válvula aórtica transcateter (TAVR) são escassos nos países em desenvolvimento.

**Objetivo:**

Avaliar a LC da TAVR no Brasil ao longo do tempo.

**Métodos:**

Analisamos dados do registro brasileiro de TAVR de 2008 a 2023. Pacientes de cada centro foram numerados cronologicamente em número sequencial de caso (NSC). A LC foi realizada usando um *spline* cúbico restrito ajustado para o EuroSCORE-II e o uso de próteses de nova geração. Ainda, os desfechos hospitalares foram comparados entre grupos definidos de acordo com o nível de experiência, com base no NSC: 1º ao 40º caso (experiência inicial), 41º ao 80º caso (experiência básica), 81º ao 120º caso (experiência intermediária) e 121º caso em diante (experiência alta). Análises adicionais foram conduzidas de acordo com o número de casos tratados antes de 2014 (>40 e ≤40 procedimentos). O nível de significância adotado foi p <0,05.

**Resultados:**

Foram incluídos 3194 pacientes de 25 centros. A idade média foi 80,7±8,1 anos e o EuroSCORE II médio foi 7±7,1. A análise da LC demonstrou uma queda na mortalidade hospitalar ajustada após o tratamento de 40 pacientes. Um patamar de nivelamento na curva foi observado após o caso 118. A mortalidade hospitalar entre os grupos foi 8,6%, 7,7%, 5,9%, e 3,7% para experiência inicial, básica, intermediária e alta, respectivamente (p<0,001). A experiência alta foi preditora independente de mortalidade mais baixa (OR 0,57, p=0,013 vs. experiência inicial). Centros com baixo volume de casos antes de 2014 não mostraram uma redução significativa na probabilidade de morte com o ganho de experiência, enquanto centros com alto volume de casos antes de 2014 apresentaram uma melhora contínua após o caso de número 10.

**Conclusão:**

Observou-se um fenômeno de LC para a mortalidade hospitalar do TAVR no Brasil. Esse efeito foi mais pronunciado em centros que trataram seus 40 primeiros casos antes de 2014 que naqueles que o fizeram após 2014.

## Graphical abstract

**Figura Central:**
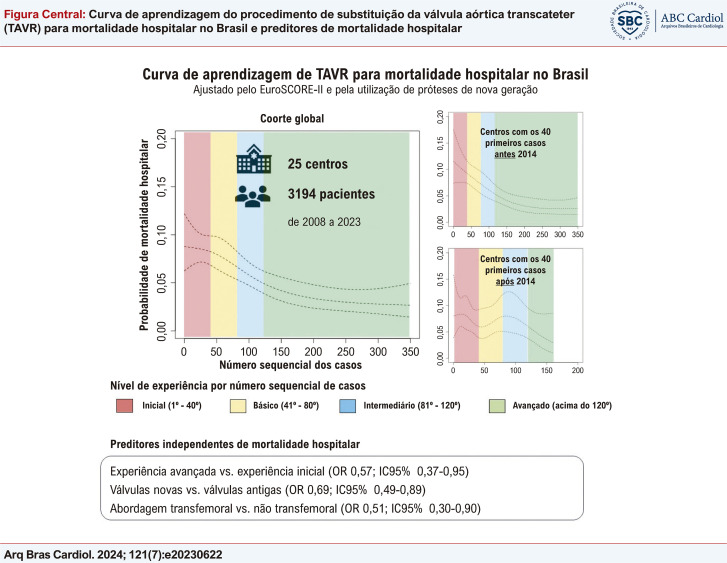
: Curva de aprendizagem do procedimento de substituição da válvula aórtica transcateter (TAVR) para mortalidade hospitalar no Brasil e preditores de mortalidade hospitalar

## Introdução

A substituição da válvula aórtica transcateter (TAVR, sigla do inglês *transcatheter aortic valve replacement*) é um procedimento multifacetado que requer um alto nível de habilidades para garantir bons desfechos clínicos. Estudos prévios demonstraram a existência de uma Curva de Aprendizagem ou *Learning Curve* (LC) e sua importância na melhoria da eficácia e da segurança do tratamento.^1-3^ No entanto, esses estudos revelaram taxas variáveis de melhoria do desfecho do paciente com o aumento da experiência, o que sugere que não existe uma LC universal para TAVR. Além disso, a maioria dos estudos avaliou dados de países norte-americanos e europeus e, uma vez que as práticas de TAVR podem variar entre diferentes locais, existem limitações em extrapolar esses dados para outras regiões.^4^

Embora em países em desenvolvimento como o Brasil tem-se observado uma taxa mais lenta de adoção da TAVR em comparação a países de alta renda,^5^ desde sua introdução em 2008, o número de procedimentos de TAVR e de centros que os realizam tem aumentado significativamente.^4^ No entanto, não existe um estudo multicêntrico nacional avaliando especificamente o comportamento da LC da TAVR ao longo da história do procedimento em países latino-americanos. O estudo possivelmente forneceria informações cruciais tanto para profissionais da saúde quanto para dirigentes, permitindo uma avaliação mais precisa das práticas locais de TAVR, alocação mais adequada de recursos e insights valiosos para melhorias contínuas da técnica. Portanto, nosso objetivo foi avaliar o impacto da LC da TAVR sobre os desfechos hospitalares desde o início do registro de TAVR no Brasil.

## Métodos

Foram utilizados dados do Registro Brasileiro de TAVR (RIBAC-NT), um registro multicêntrico nacional em andamento, desenvolvido pela Sociedade Brasileira de Hemodinâmica e Cardiologia Intervencionista (SBHCI). O protocolo inicial do registro foi publicado anteriormente.^6^ Em resumo, os centros participantes incluíram todos os procedimentos consecutivos de TAVR por meio de uma plataforma *online* com monitoramento central remoto dos dados. Em 2020, um protocolo atualizado foi submetido e aprovado pelo comitê de ética central para estender a duração do registro. Para fins da presente análise, incluímos centros com um mínimo de 25 pacientes consecutivos incluídos no registro e centros com o protocolo de registro atualizado aprovado por seus comitês de ética locais, que os dispensava da obrigatoriedade do termo de consentimento assinado pelos participantes, uma vez que o estudo impunha um risco mínimo.

Nós incluímos todos os procedimentos consecutivos de TAVR na análise final. Excluímos os casos em que a informação hospitalar não estava disponível. Usamos múltiplas imputações para manejar valores faltantes, e um modelo de pareamento preditivo médio para as variáveis numéricas, e regressão logística (logreg) para as variáveis binárias (com dois níveis). Foram conferidos os valores imputados, a distribuição residual, e os coeficientes de convergência. A maioria das variáveis estava disponível no conjunto de dados. Nós não fizemos imputação dos dados faltantes para os desfechos. A etapa de imputação resultou em cinco conjuntos completos de dados, cada um contendo diferentes estimativas dos valores faltantes para todos os pacientes da coorte. Após a imputação, nós reunimos e combinamos todos os cinco conjuntos para realizar as regressões logísticas.

### Avaliação da LC e análise estatística

**Figura 1:**
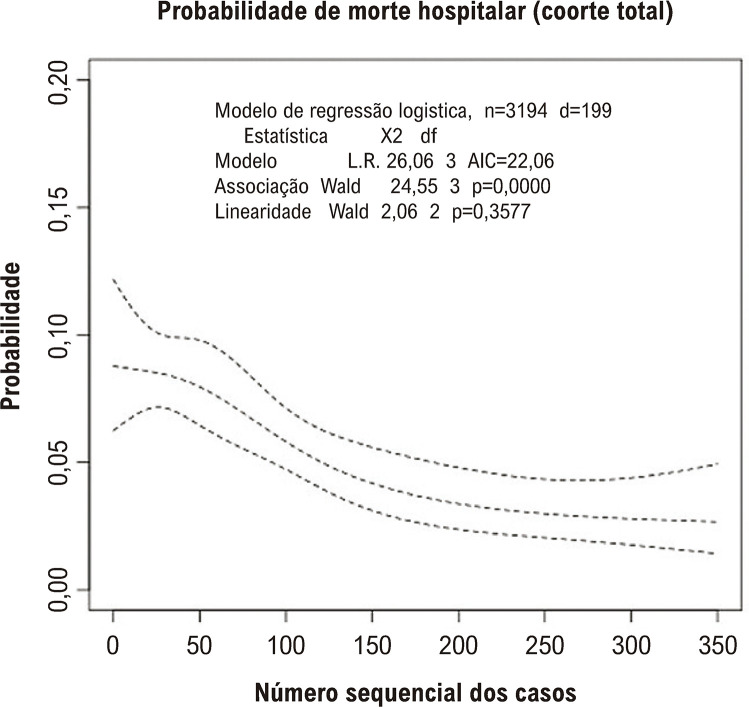
– Regressão spline para mortalidade hospitalar (ajustada para EuroSCORE-II log-transformado e a utilização de válvula cardíaca transcateter) para determinar a curva de aprendizagem da coorte global.

Seguindo um método similar publicado anteriormente para avaliar LC da TAVR em múltiplos centros,^1^os pacientes de cada centro foram numerados em sequência, cronologicamente. Para definir pontos de cortes ótimos e para determinar se houve um término da LC, nós utilizamos um *spline* cúbico restrito ajustado para o EuroSCORE-II e para o uso de Válvula Cardíaca Transcateter (VCT) de nova geração como demonstrado na Figura 1. Uma análise *grid search* foi aplicada em um intervalo de números sequenciais de casos (NSCs) (do NSC 10 ao 350), com incrementos de 1. Após a definição dos pontos de corte das sequências de casos, os NSCs subsequentes foram divididos em quartis, e a mortalidade hospitalar comparada usando um teste de regressão logística. O ponto de corte ótimo foi definido como o número de casos necessários para observar uma primeira queda significativa na curva de mortalidade (40 casos como mostrado na Figura 1). Os NSCs foram agrupados como: 1º ao 40º caso (experiência inicial), 41º ao 80º caso (experiência básica), 81º ao 120º caso (experiência intermediária) e 121º caso em diante (experiência alta). O final da LC foi determinado com base no menor valor de limite superior do intervalo de confiança de 95% do teste de regressão logística que fosse inferior ao limiar do nível de significância.

Outra análise foi realizada usando-se o ano de chegada no Brasil das VTC de mais nova geração (2014) como um ponto de corte de agrupamento. Para determinar se houve uma diferença no padrão da LC entre os centros que adotaram o procedimento de TAVR mais precocemente ou mais tardiamente, os hospitais foram divididos em dois grupos: i) aqueles que concluíram a experiência inicial (≥40 procedimentos) antes de 2014) e ii) aqueles que concluíram a experiência inicial após 2014. Nós comparamos as características basais e os desfechos hospitalares entre os grupos segundo a experiência, usando a experiência inicial como controle. O desfecho primário foi mortalidade hospitalar ou mortalidade em 30 dias para indivíduos com duração de internação maior que 30 dias. Desfechos secundários incluíram complicações vasculares, sangramento maior ou de risco à vida, e acidente vascular cerebral. Todos os desfechos foram classificados de acordo com os critérios VARC-2.^7^

As variáveis categóricas foram relatadas como número total de eventos e porcentagens. Para as variáveis contínuas, os dados com distribuição normal foram apresentados como média ± desvio padrão (DP) e os dados sem distribuição normal como mediana e Intervalo Interquartil (IIQ). A normalidade da distribuição e as variâncias foram verificadas usando histogramas e o teste de Kolmogorov-Smirnov. O teste do qui-quadrado, a análise de variância univariada (ANOVA), ou o teste de Kruskal-Wallis foi usado para comparar os dados basais e os desfechos não ajustados. O teste post-hoc de Bonferroni foi realizado quando apropriado. Nenhum teste post-hoc foi conduzido após o teste de Kruskal-Wallis. O Teste de Tendência de Cochran-Armitage (TTCA) foi usado para todas as variáveis binárias, em que a variável de dois níveis representa a resposta, e o NSC representa a variável explanatória. Regressões logísticas foram construídas para avaliar a predição dos grupos de experiência na morte hospitalar, ajustando-se quanto aos potenciais fatores de confusão (VTC de nova geração vs. geração antiga; abordagem transfemoral vs. não transfemoral, “*valve-in-valve*”, e o EuroSCORE II). As VTC de geração antiga eram aquelas da primeira linha de dispositivos comercialmente disponíveis no Brasil (Tabela S1). Outros preditores potenciais de morte hospitalar foram verificados, mas não associados com o desfecho na presente coorte: gradiente médio da válvula aórtica no eco basal, válvula aórtica bicúspide, anestesia geral, acesso percutâneo, marca da válvula, pré-dilatação com balão, e pós-dilatação com balão. Como o EuroSCORE-II inclui a maioria das variáveis da Tabela 1, essas não foram incluídas no modelo devido à colinearidade. Valores de p < 0,05 foram considerados estatisticamente significativos. As análises foram realizadas usando o R (v.3.5.3).

**Tabela 1:** – Características dos indivíduos incluídos

	1º ao 40º caso	41º ao 80º caso	81^o^ ao 120º caso	121º caso em diante	Valor p	TTCA* Valor p
Tamanho da amostra (n)	991	616	458	1129		
Idade	80,9±7,3	80,9±7,5	81,7±7	79,9±9,3	<0,001	
Sexo feminino	520 (52,5)	283 (45,9)	27 (49,6)	521 (46,1)	0,014	0,012
Diabetes	344 (34,8)	210 (34,1)	150 (32,8)	360 (32,1)	0,593	0,170
NYHA						
III	512 (51,8)	302 (49)	190 (41,8)	422 (47,3)		
IV	276 (27,9)	140 (22,7)	116 (25,5)	117 (13,1)		
Fibrilação atrial	22 (17,9)	13 (12)	20 (25)	42 (20,2)	0,133	0,255
Marca-passo	17 (8,3)	15 (7,9)	14 (8,9)	35 (9,3)	0,944	0,593
Infarto do miocárdio prévio	125 (13,5)	92 (16,3)	66 (15,8)	98 (9,6)	<0,001	0,007
ICP prévia	285 (29,1)	169 (27,9)	143 (31,3)	213 (22)	<0,001	0,002
CABG prévio	152 (5,5)	120 (19,5)	75 (16,4)	117 (12,6)	0,003	0,038
Cirurgia valvar prévia	6 (7,2)	4 (6,4)	2 (5)	14 (8,3)	0,668	0,958
Doença cerebrovascular	144 (14,8)	82 (13,6)	52 (11,4)	87 (7,8)	<0,001	<0,001
AVC prévio	71 (6,3)	36 (6)	28 (6,1)	61 (5,4)	0,335	0,089
Doença vascular periférica	164 (16,9)	112 (18,5)	82 (17,9)	97 (8,7)	<0,001	<0,001
DPOC	177 (18,3)	121 (20,0)	99 (21,7)	108 (9,6)	<0,001	<0,001
Creatinina, mg/dL	1,3±0,8	1,3±0,9	1,4±1,2	1,3±0,9	0,461	
Echo basal						
FEVE	59,1±13,9	58,9±14	59,1±12,9	58,3±13	0,005	
Gradiente médio	48±17,6	44,8±18	46,24±17,4	44±17,8	<0,001	
Válvula aórtica bicúspide	35 (5,2)	18 (3,7)	11 (3,1)	9 (3,0)	0,160	0,413
Risco cirúrgico						
EuroSCORE-II	7,71 (8,00)	7,42 (6,64)	8,14 (8,25)	5,37 (5,75)	<0,001	

*Fonte: Bernardi, 2023. Valores são n (%) ou média (±SD). TTCA: Teste de Tendência de Cochran-Armitage; ICP: intervenção coronária percutânea; CABG: bypass da artéria coronária; AVC: acidente vascular cerebral; DPOC: doença pulmonar obstrutiva crônica; FEVE: fração de ejeção do ventrículo esquerdo.*

## Resultados

Foram incluídos 3194 pacientes submetidos à TAVR em 25 centros brasileiros. Dez casos foram excluídos por falta de informação suficiente na internação. O primeiro caso foi realizado em fevereiro de 2008 e o último em fevereiro de 2023 (Figura Central). Havia 111 casos faltantes para a variável “abordagem transfemoral” e 63 para a geração (nova/antiga) da prótese, os quais foram tratados com múltiplas imputações.

### Características basais e do procedimento

As Tabelas 1 e 2 resumem as características basais e do procedimento da população geral e de cada grupo conforme nível de experiência. Ao comparar os dados basais dos pacientes entre os grupos, observou-se que os grupos com experiência inicial, básica e intermediária estavam equilibrados, com idade média similar e pequenas diferenças nas taxas de diabetes mellitus, doença arterial coronariana, doença arterial periférica e DPOC, com um EuroSCORE II médio de 7,7±8, 7,4±6.6, e 8,1±8.3 respectivamente. No grupo com experiência alta, os pacientes apresentaram menos comorbidades e um EuroSCORE II significativamente mais baixo (5,4±5,7; p=0,012, p=0,019, e p<0,001 em comparação aos grupos com experiência inicial, básica, e intermediária respectivamente).

**Tabela 2:** – Características do procedimento nos pacientes incluídos

	1º ao 40º caso	41º ao 80º caso	81^o^ ao 120º caso	121º caso em diante	Valor p	TTCA* Valor p
Tamanho amostral (n)	991	616	458	1129		
Ano em que metade dos procedimentos foram realizados	Abril/2015	Maio/2016	Janeiro/2017	Abril/2019		
VCT de nova geração	363 (36,7)	303 (49,3)	292 (63,9)	66 (90)	<0,001	<0,001
Valve-in-valve	43 (4,4)	18 (3,1)	23 (5,4)	50 (4,9)	0,271	0,333
Anestesia geral	744 (75,8)	409 (71,9)	324 (77)	401 (43,8)	<0,001	<0,001
Abordagem transfemoral	955 (96,8)	575 (93,8)	433 (95,1)	978 (96,4)		<0,001
Acesso percutâneo	707 (71,6)	516 (84,2)	405 (89,2)	982 (96,9)	<0,001	<0,001
Pré-dilatação com balão	406 (42,2)	257 (43,4)	184 (40,7)	368 (37,0)	0,042	0,012
MARCAS DAS VÁLVULAS						
Sapien XT	188 (19)	134 (21,8)	81 (17,7)	55 (5,1)		
CoreValve	413 (41,8)	157 (25,6)	53 (11,6)	28 (2,6)		
Lotus	15 (1,5)	3 (0,5)	28 (6,1)	16 (1,5)		
Sapien S3	179 (18,1)	112 (18,2)	126 (27,6)	528 (49,2)		
Evolut R/PRO	30 (13,2)	167 (27,2)	141 (30,9)	251 (23,4)		
Braile	9 (0,9)	17 (2,8)	3 (0,7)	8 (0,7)		
Portico	11 (1,1)	0 (0)	0 (0)	8 (0,7)		
Acurate Neo/Neo2	41 (4,1)	16 (2,6)	17 (3,7)	146 (13,6)		
Myval	0 (0)	3 (0,5)	6 (1,3)	12 (1,1)		
Não informado	2 (0,2)	5 (0,8)	2 (0,4)	21 (2)		
Pós-dilatação com balão	295 (30,8)	173 (31,1)	125 (29,8)	255 (28,1)	0,557	0,198
Embolização da válvula	25 (2,7)	13 (2,7)	8 (2,2)	10 (1,1)	0,070	0,013
Necessidade de 2ª válvula	27 (2,9)	19 (4)	9 (2,5)	11 (1,2)	0,010	0,008
Oclusão da artéria coronária	4 (0,5)	6 (1,4)	4 (1,2)	1 (0,1)	0,015	0,197
Ruptura de anel	8 (0,9)	1 (0,2)	1 (0,3)	5 (0,5)	0,371	0,407
Tamponamento	34 (3,7)	14 (3,1)	13 (3,9)	19 (2,3)	0,345	0,071
Cirurgia aberta de conversão	34 (3,5)	12 (2,0)	10 (2,2)	11 (1,0)	0,001	<0,001

*Fonte: Bernardi, 2023. Valores são n (%); VCT: válvula cardíaca transcateter; TTCA: Teste de Tendência de Cochran-Armitage.*

A maioria dos procedimentos foi transfemoral (96%), sem diferença significativa entre os grupos. No entanto, à medida que a experiência aumentava, uma maior proporção de casos foi tratada com uma abordagem percutânea, sem anestesia geral. A técnica *valve-in-valve* correspondeu a somente 4,4% dos procedimentos, com taxas similares entre os grupos. O uso de VCT de mais nova geração aumentou consistentemente à medida em que a experiência dos centros aumentou, de 36,7% a 90% nos centros com experiência inicial e alta, respectivamente. Embolização da válvula, necessidade de uma segunda válvula, obstrução coronária, e conversão para cirurgia aberta foram menos comuns nos centros com alta experiência (p<0,05 para todos).

### Curva de aprendizagem e desfechos

A Figura 1 ilustra a LC da TAVR com uma regressão *spline* para mortalidade hospitalar ajustada para o EuroSCORE II com transformação log e para o uso de VCT de mais nova geração, mostrando que foi necessário tratar 40 casos até a ocorrência da primeira queda na probabilidade ajustada de morte. Uma mudança na inclinação foi observada no NSC118, sinalizando uma estabilização da mortalidade hospitalar após esse nível de experiência. Estabeleceu-se que o final da LC tenha ocorrido no NSC303 com base no menor limite superior do intervalo de confiança de 95% do teste de regressão logística que estava abaixo do nível de significância.

**Figura 2:**
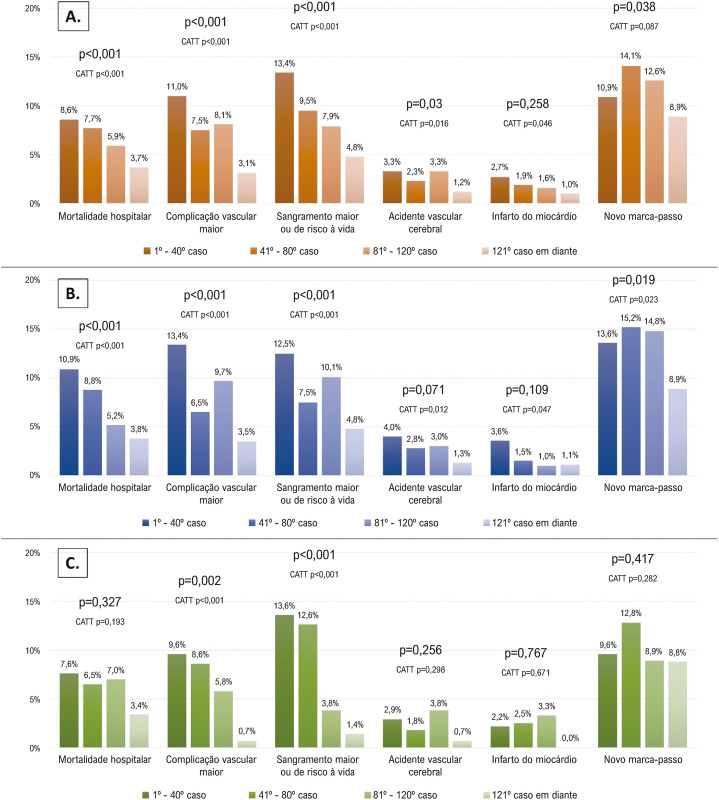
– Desfechos hospitalares após substituição da válvula aórtica transcateter (TAVR) segundo o número sequencial de casos; A) população geral; B) centros com alto volume de procedimentos de TAVR [≥40 procedimentos] antes de 2014. C) centros com baixo volume de procedimentos de TAVR [<40 procedimentos] antes de 2014; TTCA: Teste de Tendência de Cochran-Armitage.

**Figura 3:**
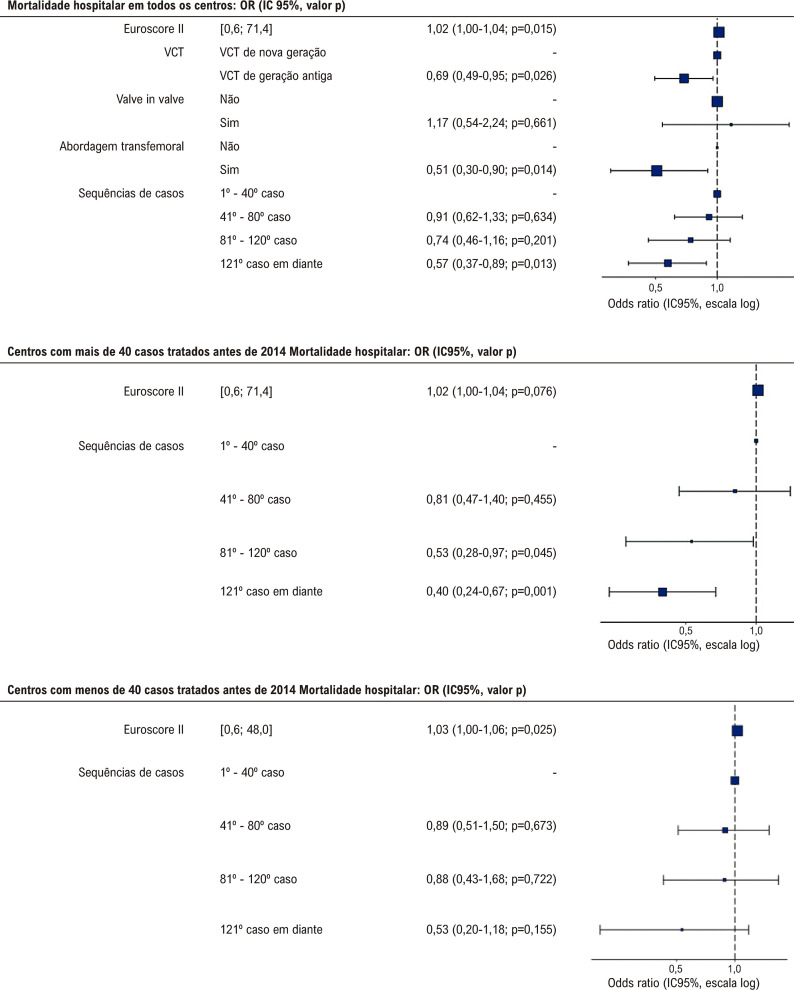
– Forest plots para taxa de mortalidade hospitalar de acordo com as faixas de sequência de casos nos centros de substituição da válvula aórtica transcateter (TAVR) com um volume maior (>40 casos) e nos centros com um volume menor (<40 casos) de pacientes tratados antes de 2014.

Ao comparar os grupos de experiência, observamos uma queda contínua na mortalidade hospitalar não ajustada a partir do grupo com experiência inicial (8,7%), básica (8%), intermediária (6,1%), e alta (4,0%) (p< 0,001) (Figura 2). Além disso, observou-se uma diferença significativa na incidência de complicações vasculares maiores, sangramento maior ou de risco à vida, e acidente vascular cerebral. Após ajuste quanto aos fatores de confusão e usando o grupo experiência inicial como controle, somente o grupo com alta experiência foi associado com uma redução significativa na mortalidade hospitalar (OR 0,52, p=0,002). A abordagem transfemoral (OR 0,51, p=0,014) e o uso de VCT de nova geração também foram preditores de uma mortalidade hospitalar mais baixa (OR 0,69, p=0,029), além de um EuroSCORE II mais baixo (Figura 3).

### Centros com experiência inicial antes e após 2014

Oito dos 25 centros concluíram a experiência inicial (primeiros 40 casos de TAVR) antes de 2014, correspondendo a 1916 pacientes com um número mediano de procedimentos por centro de 222 (IIQ 163-282). Os demais 17 centros completaram a experiência inicial após 2014, correspondendo a 1278 pacientes e um número mediano de procedimentos por centro de 56 (IIQ 34,5-115). As Tabelas suplementares S1 e S2 descrevem as características basais e do procedimento dos grupos (por nível de experiência) e por sequências de casos nos centros com experiência inicial antes de 2014 e após 2014. Em geral, os pacientes apresentaram escores de risco similares, segundo o EuroSCORE II (média de 7,0±7,5 e 6,9±6,8 para experiência inicial antes e após 2014, respectivamente). Nos centros com experiência inicial após 2014, VCTs de nova geração foram implantadas com uma frequência significativamente mais alta (70,3% vs. 55,4%, p<0,001).

**Figura 4:**
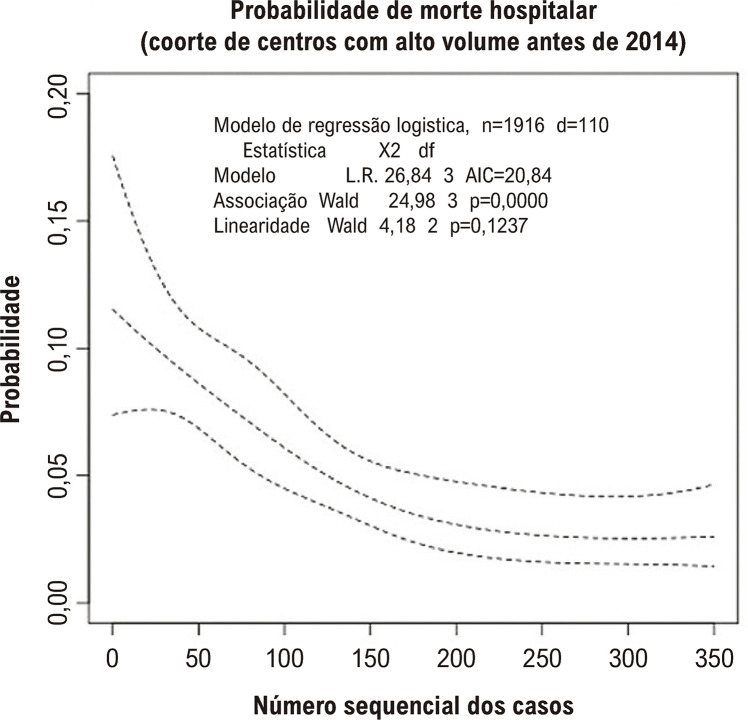
– Regressão spline para mortalidade hospitalar (ajustada para EuroSCORE-II log-transformado e o uso de válvula cardíaca transcateter) para determinar a curva de aprendizagem dos centros com experiência inicial antes de 2014.

**Figura 5:**
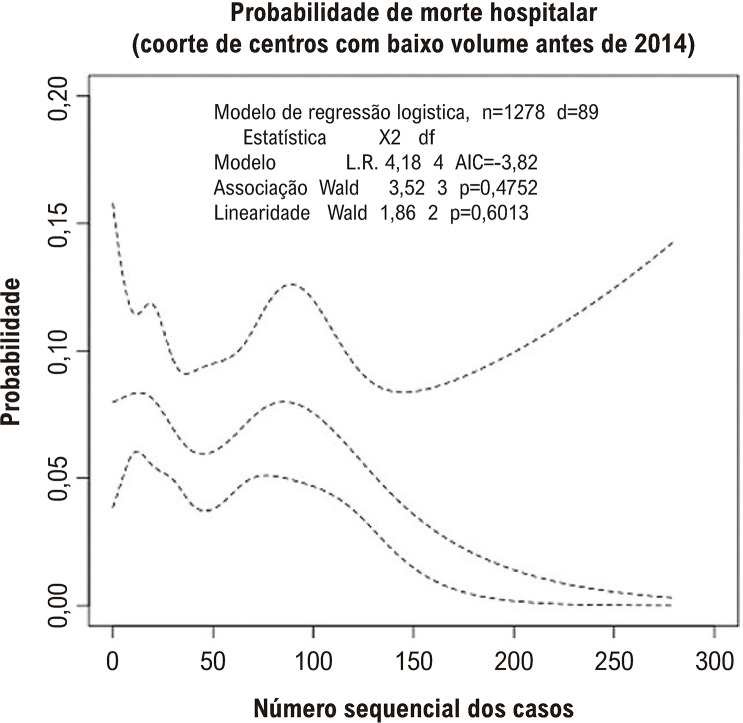
– Regressão spline para mortalidade hospitalar (ajustada para EuroSCORE-II log-transformado e o uso de válvula cardíaca transcateter) para determinar a curva de aprendizagem dos centros com experiência inicial após 2014.

Como mostrado na Figura 4, a LC das duas coortes apresentaram diferentes padrões. Entre os centros com experiência inicial concluída antes de 2014, observou-se um padrão típico de LC, com uma queda inicial da mortalidade ocorrendo precocemente, após os primeiros dez casos. Uma mudança na inclinação foi observada no CSN81, sinalizando uma estabilização da mortalidade após esse nível de experiência. Já entre os centros com experiência inicial concluída após 2014 (Figura 5), observamos, inicialmente, uma mortalidade mais baixa, mas a curva manteve-se estável até aproximadamente o NCS100. Depois disso, a curva caiu, embora com um alargamento do intervalo de confiança devido ao menor número de centros com mais de 100 casos nessa coorte.

Na coorte de centros que adotaram a TAVR antes de 2014, experiência intermediária e experiência alta apresentaram uma razão de chances (*odds ratio*) de 0,47 (p=0,027) e 0,44 (p-0,003), respectivamente, para mortalidade hospitalar, em comparação à experiência inicial após ajuste para o EuroSCORE II. Não encontramos uma relação significativa entre experiência adquirida e mortalidade hospitalar entre os centros que adotaram a TAVR mais tardiamente (após 2014) (Figura 2). A Figura 2 mostra as taxas de eventos não ajustados das duas coortes de acordo com os diferentes níveis de experiência.

## Discussão

Neste amplo registro nacional de TAVR no Brasil, nós avaliamos a LC analisando o impacto da experiência acumulada dos centros sobre a mortalidade hospitalar, e os resultados principais foram: 1) Vinte e cinco centros e 3194 pacientes foram incluídos; a experiência acumulada foi associada a uma redução da mortalidade hospitalar, com a LC mostrando uma primeira queda na mortalidade a partir do caso número 40 com uma estabilização da curva a partir do caso número 118; 2) alta experiência, determinada por uma experiência acumulada de mais de 120 casos, foi um preditor independente de mortalidade hospitalar mais baixa, e se associou com taxas mais baixas não ajustadas de complicações, como sangramento clinicamente relevante, complicações vasculares maiores, e acidente vascular cerebral; 3) houve dois padrões distintos de LC nos centros que tiveram sua experiência inicial antes e após 2014, sugerindo uma possível atenuação da relação entre experiência acumulada e menor mortalidade hospitalar nos centros que iniciaram seus programas de TAVR mais tardiamente; 4) além do EuroSCORE II, a abordagem transfemoral e o uso de VCTs de nova geração foram variáveis independentes associadas com mortalidade hospitalar mais baixa.

O conhecimento da LC em procedimentos complexos e de alto custo como a TAVR é crucial para os processos de planejamento que visam a melhoria contínua das práticas clínicas e a otimização de alocação de recursos futuros. Embora a LC da TAVR tenha sido avaliada em estudos anteriores,^1-3,8-10^ nosso estudo é o primeiro estudo multicêntrico conduzido em um país da América do Sul, cuja realidade é bem distinta da de países de alta renda. No Brasil e em outras nações em desenvolvimento, o acesso da população à TAVR tem sido significativamente restrito,^4,5^ devido principalmente a restrições econômicas e acesso limitado ao procedimento no sistema público de saúde.^11,12^ Por exemplo, até 2017, foram realizados menos de 10 procedimentos de TAVR por milhão de habitantes no Brasil, em comparação a mais de 100-150 em países como os EUA, França e Alemanha.^5^ Essa variação regional na acessibilidade à TAVR e no volume de procedimentos pode ser um fator determinante da LC em um país. Em uma nação continental como o Brasil, uma análise incorporando instituições de várias regiões do país é de suma importância.

Nossos dados são consistentes com os de estudos multicêntricos internacionais que demonstraram um declínio na incidência de eventos adversos precoces à medida que as instituições ganharam experiência.^1-3,8^ Um dado importante foi que observamos uma diminuição na mortalidade a partir do caso 40, similar a Russo et al.^1^ que também encontraram uma melhora a partir do caso 38. Contudo, esses achados contrastam com os do estudo de Wassef et al.,^2^ que encontraram uma melhora a partir do caso 75; ambos foram grandes estudos multicêntricos, conduzidos principalmente em instituições de países de alta renda. Essa melhora na mortalidade estendeu-se além do procedimento número 120 em nosso estudo, e além dos casos 170 e 150 nos estudos de Russo et al.^1^ e de Wassef et al.,^2^ respectivamente. Apesar das distintas realidades, esses resultados demonstram que a LC geral no Brasil foi, de certa forma, comparável à de países de alta renda com acesso bem mais amplo à TAVR, refletindo que a técnica do procedimento possui uma LC intrínseca relacionada à proficiência do operador.

No entanto, é essencial reconhecer que a TAVR evoluiu desde sua criação. Nos últimos 15 anos, novas versões de dispositivos e técnicas refinadas, incluindo abordagens menos invasivas, surgiram. Destaca-se um estudo que avaliou o desenvolvimento de TAVR em centros da América Latina, a maioria deles no Brasil, revelando uma mudança significativa na prática entre 2015 e 2020, com maior incorporação de procedimentos minimalistas e adoção universal das versões mais recentes de VCTs.^4^ Para determinar se essas mudanças temporais na TAVR afetaram a LC, nós conduzimos uma subanálise, dividindo centros com experiência inicial (primeiros 40 casos tratados) antes de 2014 daqueles com experiência inicial completada após 2014, ano em que a primeira VCT de nova geração tornou-se comercialmente disponível no Brasil. Interessante notar que, o fenômeno da LC, com uma redução inicial da mortalidade hospitalar, foi praticamente ausente entre os centros que iniciaram a TAVR mais tardiamente, diferentemente dos centros que a adotaram mais cedo, em que se observou uma nítida LC inicial, com uma redução na probabilidade de morte já ocorrendo após os primeiros dez casos. Esses dados são similares aos de Russo et al.,^1^ que analisaram dados do registro TVT norte-americano, e não encontraram evidência de uma LC em centros em que a experiência inicial ocorreu após 2015, com a prótese com balão expansível Sapien S3 (Edwards Lifesciences, Irvine, CA). Esses centros não apresentaram uma melhora significativa nos desfechos clínicos com o aumento da experiência, e seus desfechos clínicos iniciais já eram comparáveis aos dos centros com maior experiência. Segundo os autores, esse resultado não foi surpreendente, uma vez que a melhoria nos dispositivos, programas de *proctoring*, e disseminação do conhecimento podem resultar em uma adoção mais rápida de técnicas de TAVR entre centros mais novos e de menor volume.^1^

Muitos fatores na prática atual podem haver contribuído para esse início com melhores resultados nos centros mais novos de TAVR, como: 1) maior conhecimento geral por toda comunidade científica e acesso a um acúmulo importante de evidência científica ao longo da história da TAVR;^13^ 2) experiência crescente e amplo acesso ao uso de VCTs mais modernas, simples, seguras e confiáveis, o que por si só propicia a melhores desfechos clínicos;^14-16^ 3) o trabalho intenso da comunidade científica, em colaboração com a indústria, para melhorar as técnicas de implantes das próteses, combinado com extensos esforços para disseminação do conhecimento;^17^ 4) melhor seleção dos pacientes, com maior inclusão de pacientes com risco cirúrgico mais baixo, além do refinamento das técnicas de imagem para melhor planejamento do procedimento;^17^ 5) programas de *proctoring* extensivos oferecidos pelas empresas das VCTs aos centros de TAVR.

No entanto, embora não tenhamos encontrado uma associação significativa entre redução de mortes hospitalares com o ganho de experiência acumulada dos centros que iniciaram TAVR mais tardiamente, parece existir uma tendência de melhora, especialmente após os primeiros 120 casos. Vale notar que somente quatro das 17 instituições possuíam pacientes no grupo de alta experiência em nosso estudo, acarretando em intervalos de confiança amplos da LC em razão do número reduzido de pacientes nesse estrato.

Portanto, não podemos descartar a possibilidade de um erro tipo II em nossa observação nessa coorte. Em uma LC típica, esperaríamos melhora dos resultados mais precocemente. Mas em nosso caso, precisamos também considerar a possibilidade de um padrão de LC com começo mais lento de progressão, atingindo a proficiência mais tardiamente nesses centros cuja experiência inicial da TAVR se deu após 2014. Apesar desses centros terem iniciado com uma taxa de mortalidade hospitalar mais baixa (7,6% vs. 10,9% dos centros que completaram sua experiência inicial antes de 2014), houve pouca ou nenhuma melhora até o caso 120. No entanto, mesmo lidando com pacientes de risco moderado a alto (EuroSCORE II médio de quase oito), a taxa de mortalidade hospitalar foi notavelmente mais alta que àquela relatada em outros estudos internacionais. Por exemplo, em um estudo do registro TVT, as instituições que começaram a trabalhar com a prótese balão expansível Sapien S3 após 2015 mantiveram-se consistentemente com uma taxa de mortalidade baixa de aproximadamente 4%, mesmo mesmo também tratando pacientes de risco intermediário a alto (escore STS médio de 7,3%).^1^ Portanto, a falta de melhora imediata à medida que os números de casos aumentaram em nossa análise sugere que essas instituições de TAVR mais novas devem ter passado por um processo de aprendizagem inicialmente mais lento. Além disso, a ocorrência relativamente alta de complicações vasculares nessa coorte, o que é frequentemente vista como um marcador de expertise de TAVR, corrobora essa hipótese.

Uma pergunta que permanece é por que o processo de aprendizagem desses centros foi atrasado em termos de redução na mortalidade hospitalar. Uma possível explicação pode ser o volume limitado de TAVR realizada nessas instituições. Antes do caso 120, o número mediano anual de procedimentos foi 6,4 (IIQ 5 a 11) em comparação a 15 (IIQ 12,3 a 17,1) para a coorte de centros que tiveram sua experiência inicial antes de 2014. Estudos prévios demonstraram consistentemente uma associação significativa entre o volume de procedimentos e a melhora de desfechos, incluindo mortalidade em curto prazo.^2,18^ Contudo, nosso estudo não foi delineado para avaliar o impacto nos desfechos clínico do volume de procedimentos dos centros, uma vez que essa análise seria extremamente complexa para ser interpretada considerando que muitos hospitais iniciaram seus programas de TAVR durante o período analisado, e a maiores deles operando com volumes limitados de casos (<20 TAVR/ano), tornando-se um desafio estatístico analisar tal associação. Dada a inclusão iminente do procedimento no sistema de saúde público brasileiro, esta é uma informação de alta relevância e deve ser devidamente considerada em futuros estudos para o contínuo avanço da TAVR no país. Portanto, é de suma importância continuar com a análise detalhada dos dados provenientes do registro brasileiro, bem como de outros países de renda mais baixa. Essa investigação aprofundada é essencial para examinar os diversos fatores associados à LC no contexto específico desses países, garantindo assim melhores resultados e avanços significativos no tratamento da TAVR em pacientes com estenose aórtica.

### Limitações do estudo

Este é um estudo observacional com dados de um registro de vida real com desfechos reportados localmente, sem uma adjudicação central. Embora desfechos padronizados pelos critérios VARC-2 tenham sido utilizados, podem existir inconsistências em seus relatos. Esse foi o motivo pelo qual escolhemos mortalidade hospitalar por todas as causas para a análise primária, para mitigar potenciais vieses de avaliação. Ainda não foi avaliado o impacto da LC sobre desfechos clínicos a médio e longo prazo, uma vez que avaliamos somente eventos intra hospitalares. Além disso, apesar de havermos ajustado o desfecho primário para o EuroSCORE II para ajustar ao número crescente de paciente de risco mais baixo tratados ao longo do tempo no registro ainda é importante considerar o potencial impacto das variáveis não medidas que possam atuar como fatores de confusão. Quanto à nossa subanálise, a maioria dos centros que adotaram a TAVR tardiamente estavam próximos de sua experiência inical, o que pode haver contribuído para a falta de um declínio significativo na mortalidade e, assim, o efeito da experiência pode ser sido imperceptível. Finalmentem a TAVR continua a evoluir rapidamente com o desenvolvimento de técnicas mais refinadas e dispositivos superiores quase que anualmente. Portanto, nossos achados podem não representar a prática mais atualizada.

## Conclusão

Ao longo da história da TAVR no Brasil, a experiência acumulada do procedimento das principais instituições no país associada a uma associou-se com uma redução na mortalidade hospitalar, indicando um fenômeno legítimo de LC. No entanto, essa relação entre experiência e melhora dos resultados foi mais impactante nos primeiros anos da história da TAVR no país. Nos centros que iniciarem seus programas de TAVR mais recentemente, apesar de terem obtido resultados clínicos iniciais mais promissores, houve pouca evolução dos seus resultados com o ganho progressivo de experiência.Esses achados contribuem para o entendimento da LC na TAVR e fornece *insights* para pesquisa futura nesse campo em rápida evolução.

## References

[1] Russo MJ, McCabe JM, Thourani VH, Guerrero M, Genereux P, Nguyen T (2019). Case Volume and Outcomes After TAVR With Balloon-Expandable Prostheses: Insights From TVT Registry. J Am Coll Cardiol.

[2] Wassef AWA, Rodes-Cabau J, Liu Y, Webb JG, Barbanti M, Muñoz-García AJ (2018). The Learning Curve and Annual Procedure Volume Standards for Optimum Outcomes of Transcatheter Aortic Valve Replacement: Findings From an International Registry. JACC Cardiovasc Interv.

[3] Minha S, Waksman R, Satler LP, Torguson R, Alli O, Rihal CS (2016). Learning Curves for Transfemoral Transcatheter Aortic Valve Replacement in the PARTNER-I Trial: Success and Safety. Catheter Cardiovasc Interv.

[4] Bernardi FLM, Ribeiro HB, Nombela-Franco L, Cerrato E, Maluenda G, Nazif T (2022). Recent Developments and Current Status of Transcatheter Aortic Valve Replacement Practice in Latin America - The WRITTEN LATAM Study. Arq Bras Cardiol.

[5] Pilgrim T, Windecker S (2018). Expansion of Transcatheter Aortic Valve Implantation: New Indications and Socio-economic Considerations. Eur Heart J.

[6] Brito FS, Carvalho LA, Sarmento-Leite R, Mangione JA, Lemos P, Siciliano A (2015). Outcomes and Predictors of Mortality After Transcatheter Aortic Valve Implantation: Results of the Brazilian Registry. Catheter Cardiovasc Interv.

[7] Kappetein AP, Head SJ, Généreux P, Piazza N, van Mieghem NM, Blackstone EH (2012). Updated Standardized Endpoint Definitions for Transcatheter Aortic Valve Implantation: The Valve Academic Research Consortium-2 Consensus Document. Eur Heart J.

[8] Lunardi M, Pesarini G, Zivelonghi C, Piccoli A, Geremia G, Ariotti S (2016). Clinical Outcomes of Transcatheter Aortic Valve Implantation: From Learning Curve to Proficiency. Open Heart.

[9] Siqueira DA, Abizaid AAC, Ramos AA, Jeronimo AD, LeBihan D, Barreto RB (2014). Impacto da Curva de Aprendizado na Seleção de Pacientes e nos Resultados Clínicos do Implante por Cateter de Prótese Aórtica. Rev Bras Cardiol Invasiva.

[10] O'Brien SM, Cohen DJ, Rumsfeld JS, Brennan JM, Shahian DM, Dai D (2016). Variation in Hospital Risk-Adjusted Mortality Rates Following Transcatheter Aortic Valve Replacement in the United States: A Report From the Society of Thoracic Surgeons/American College of Cardiology Transcatheter Valve Therapy Registry. Circ Cardiovasc Qual Outcomes.

[11] Bergmann T, Sengupta PP, Narula J (2017). Is TAVR Ready for the Global Aging Population?. Glob Heart.

[12] Lopes MACQ, Nascimento BR, Oliveira GMM (2020). Treatment of Aortic Stenosis in Elderly Individuals in Brazil: How Long Can We Wait?. Arq Bras Cardiol.

[13] Güzel T, Arslan B (2022). Examination of the Most Cited Studies on Transcatheter Aortic Valve Replacement with Bibliometric Analysis. Echocardiography.

[14] Sattar Y, Prakash P, Almas T, Mir T, Titus A, Ahmad S (2023). Cardiovascular Outcomes of Older versus Newer Generation Transcatheter Aortic Valve Replacement Recipients: A Systematic Review & Meta-analysis. Curr Probl Cardiol.

[15] Wendler O, Schymik G, Treede H, Baumgartner H, Dumonteil N, Ihlberg L (2017). SOURCE 3 Registry: Design and 30-Day Results of the European Postapproval Registry of the Latest Generation of the SAPIEN 3 Transcatheter Heart Valve. Circulation.

[16] Falk V, Baumgartner H, Bax JJ, De Bonis M, Hamm C, Holm PJ (2017). 2017 ESC/EACTS Guidelines for the Management of Valvular Heart Disease. Eur J Cardiothorac Surg.

[17] Spears J, Al-Saiegh Y, Goldberg D, Manthey S, Goldberg S (2020). TAVR: A Review of Current Practices and Considerations in Low-Risk Patients. J Interv Cardiol.

[18] Vemulapalli S, Carroll JD, Mack MJ, Li Z, Dai D, Kosinski AS (2019). Procedural Volume and Outcomes for Transcatheter Aortic-Valve Replacement. N Engl J Med.

