# Supplementary material for: Curva de Aprendizagem da Mortalidade Hospitalar da Substituição da Válvula Aórtica Transcateter: Insights do Registro Nacional Brasileiro
Source: Arq Bras Cardiol. 2024 Jul 10;121(7):e20230622. [Article in Portuguese] doi: 10.36660/abc.20230622 (PMC12080715; doi:10.36660/abc.20230622)
Supplement: Translation

# Translation: Learning Curve for In-Hospital Mortality of Transcatheter Aortic Valve Replacement: Insights from the Brazilian National Registry

**DOI:** 10.36660/abc.20230622i

**Authors:** Fernando Luiz de Melo Bernardi, Alexandre A. Abizaid, Fábio Sândoli de Brito, Pedro A. Lemos, Dimytri Alexandre Alvim de Siqueira, Ricardo Alves Costa, Rogério Eduardo Gomes Sarmento Leite, Fernanda Marinho Mangione, Luiz Eduardo Koenig São Thiago, José A. Mangione, Valter Correia de Lima, Adriano Dourado Oliveira, Marcos Antônio Marino, Carlos José Francisco Cardoso, Paulo R. A. Caramori, Rogério Tumelero, Antenor Lages Fortes Portela, Mauricio Prudente, Leônidas Alvarenga Henriques, Fabio Solano Souza, Cristiano Guedes Bezerra, Guy F. A. Prado, Leandro Zacaris Figueiredo Freitas, Ederlon Ferreira Nogueira, George César Ximenes Meireles, Renato Bastos Pope, Enio Guerios, Pedro Beraldo de Andrade, Luciano de Moura Santos, Mauricio Felippi de Sá Marchi, Nelson Henrique Fantin Fundão, Henrique Barbosa Ribeiro

**Affiliations:** 1 Brazil Instituto do Coração do Hospital das Clínicas da Faculdade de Medicina da Universidade de São Paulo, São Paulo, SP – Brazil; 2 Brazil Hospital Sírio-Libanês, São Paulo, SP – Brazil; 3 Brazil Hospital Israelita Albert Einstein, São Paulo, SP – Brazil; 4 Brazil Instituto Dante Pazzanese de Cardiologia, São Paulo, SP – Brazil; 5 Brazil Instituto de Cardiologia, Porto Alegre, RS – Brazil; 6 Brazil Hospital Beneficência Portuguesa de São Paulo, São Paulo, SP – Brazil; 7 Brazil Hospital SOS Cárdio, Florianópolis, SC – Brazil; 8 Brazil Santa Casa de Misericórdia de Porto Alegre, Porto Alegre, RS – Brazil; 9 Brazil Hospital Santa Izabel, Salvador, BA – Brazil; 10 Brazil Hospital Madre Teresa, Belo Horizonte, MG – Brazil; 11 Brazil Hospital Naval Marcilio Dias, Rio de Janeiro, RJ – Brazil; 12 Brazil Hospital São Lucas da PUCRS, Porto Alegre, RS – Brazil; 13 Brazil Universidade de Passo Fundo, Passo Fundo, RS – Brazil; 14 Brazil Associação Piauiense de Combate ao Câncer, Teresina, PI – Brazil; 15 Brazil Hospital Encore, Goiânia, GO – Brazil; 16 Brazil Hospital Albert Sabin, Juiz de Fora, MG – Brazil; 17 Brazil Hospital Universitário Professor Edgard Santos, Salvador, BA – Brazil; 18 Brazil Hospital Cardio-Pulmonar, Salvador, BA – Brazil; 19 Brasil Hospital Aliança Rede D´Or, Salvador, BA – Brasil; 20 Brasil Universidade Federal de Goiás, Goiânia, GO – Brasil; 21 Brazil Hospital do Coração de Londrina, Londrina, PR – Brazil; 22 Brazil Instituto de Assistência Médica ao Servidor Público Estadual de São Paulo, São Paulo, SP – Brazil; 23 Brasil Hospital Hans Dieter Schmidt, Joinville, SC – Brasil; 24 Brazil Universidade Federal do Paraná – Hospital de Clinicas, Curitiba, PR – Brazil; 25 Brazil Santa Casa de Misericórdia de Marília, Marilia, SP – Brazil; 26 Brazil Hospital Santa Lúcia, Brasília, DF – Brazil

**Keywords:** Transcatheter Aortic Valve Replacement, Aortic Valve Stenosis, Hospital Mortality

## Abstract

**Background:**

Robust data on the learning curve (LC) of transcatheter aortic valve replacement (TAVR) are lacking in developing countries.

**Objective:**

To assess TAVR’s LC in Brazil over time.

**Methods:**

We analyzed data from the Brazilian TAVR registry from 2008 to 2023. Patients from each center were numbered chronologically in case sequence numbers (CSNs). LC was performed using restricted cubic splines adjusted for EuroSCORE-II and the use of new-generation prostheses. Also, in-hospital outcomes were compared between groups defined according to the level of experience based on the CSN: 1^st^ to 40^th^ (initial-experience), 41^st^ to 80^th^ (early-experience), 81^st^ to 120^th^ (intermediate-experience), and over 121^st^ (high-experience). Additional analysis was performed grouping hospitals according to the number of cases treated before 2014 (>40 and ≤40 procedures). The level of significance adopted was <0.05.

**Results:**

A total of 3,194 patients from 25 centers were included. Mean age and EuroSCORE II were 80.7±8.1 years and 7±7.1, respectively. LC analysis demonstrated a drop in adjusted in-hospital mortality after treating 40 patients. A leveling off of the curve was observed after case #118. In-hospital mortality across the groups was 8.6%, 7.7%, 5.9%, and 3.7% for initial-, early-, intermediate-, and high-experience, respectively (p<0.001). High experience independently predicted lower mortality (OR 0.57, p=0.013 vs. initial experience). Low-volume centers before 2014 showed no significant decrease in the likelihood of death with gained experience, whereas high-volume centers had a continuous improvement after case #10.

**Conclusion:**

A TAVR LC phenomenon was observed for in-hospital mortality in Brazil. This effect was more pronounced in centers that treated their first 40 cases before 2014 than those that reached this milestone after 2014.

## Introduction

Transcatheter aortic valve replacement (TAVR) is a multifaceted procedure that requires high-level of skills to ensure good clinical outcomes. Prior studies have demonstrated the existence of a learning curve (LC) and its importance in improving treatment efficacy and safety.^1-3^ However, these studies revealed differing rates of patient outcome improvement with increasing experience, suggesting that a universal LC for TAVR does not exist. Also, most studies evaluated data derived from North American and European countries and, as TAVR practices may vary among different locations, limitations exist in extrapolating these data other regions.^4^

Developing nations like Brazil have seen a slower rate of TAVR adoption in comparison to high-income countries.^5^ Yet, since its introduction in 2008, the number of TAVR procedures and centers performing the procedure have increased significantly.^4^ Nonetheless, there is no nationwide multicenter study specifically evaluating the behavior of TAVR LC throughout the history of the procedure in Latin American countries. It would possibly provide crucial information for both healthcare professionals and policymakers to evaluate TAVR practices, allocate resources appropriately, and valuable insights for ongoing improvement. Therefore, our aim was to evaluate the impact of the TAVR LC for in-hospital outcomes since the beginning of the Brazilian nationwide TAVR registry.

## Methods

Data from the Brazilian TAVR Registry (RIBAC-NT), an ongoing nationwide multicenter registry developed by the Brazilian Society of Hemodynamics and Interventional Cardiology (SBHCI), were used. The initial protocol of the registry has been previously published.^6^ Briefly, participating centers included all consecutive TAVR procedures through an online platform with remote central data monitoring. In 2020, an updated protocol was submitted and approved by the central ethical committee to extend the duration of the registry. For the purpose of the present analysis, we have included centers with a minimum of 25 consecutive patients in the registry and those with the updated registry protocol approved by their local Ethics Committee, which contained a waiver of informed consent of the included patients, as the study posed minimal risk.

We included all consecutive TAVR procedures in the final analyses. We excluded cases where in-hospital information was not available. We used multiple imputations to handle missing values, and a predictive mean matching model for numeric variables, and logistic regression (logreg) for binary variables (with two levels). Imputed values, residual distribution, and convergence coefficients were checked. Most variables were available in the dataset. We did not impute missing values for the outcomes. The imputation step resulted in five complete data sets, each of them containing different estimates of the missing values for all patients in the cohort. After imputation, we pooled and merged all five datasets to perform logistic regressions.

### Learning curve assessment and statistical analysis

Following a similar method previously described to assess the LC of TAVR in multiple centers,^1^ patients from each center were sequentially numbered chronologically. To define optimal cut-off points and to determine if there was an end of the LC, we used restricted cubic splines adjusted for EuroSCORE-II and a new-generation transcatheter heart valve (THV) as shown in Figure 1. A grid search analysis was applied across a range of case sequence numbers (CS#) from 10 to 350 by increments of 1. After the case sequence cutoffs were established, subsequent CS#s were divided into quartiles, and in-hospital mortality was compared using a logistic regression test. The optimal cut-off point was defined as number of cases necessary to observe a first significant drop in mortality curve (40 cases as shown in Figure 1). The CS#s were grouped as: 1^st^ to 40^th^ case (initial-experience), 41^st^ to 80^th^ case (early-experience), 81^st^ to 120^th^ case (intermediate-experience) and over 121^st^ case (high-experience). The end of the LC was determined based on the smallest upper bound of the 95% confidence interval of the logistic regression test that was below the significance level threshold.

**Figure 1:** – Spline regressions for in-hospital death (adjusted to log-transformed EuroSCORE-II and the utilization of new generation THV) to determine the learning curve of the global cohort.

Additional analysis was performed using the year of arrival of newer-generation THV in Brazil (2014) as a grouping cut-off. To determine whether there was a difference in the pattern of the LC between early and late TAVR adopters, hospitals were divided into two groups: i) those who had completed their initial experience before 2014 (≥40 procedures before 2014) and ii) those who had completed their initial experience after 2014. We compared the baseline characteristics and in-hospital outcomes between the experience groups, using the group with initial experience as control. The primary outcome was in-hospital mortality, with 30-day mortality assessed for hospitalizations lasting longer than 30 days. Key secondary outcomes included major vascular complications, major or life-threatening bleeding, and any stroke. All outcomes were classified according to the VARC-2 criteria.^7^

Categorical variables were reported as total numbers of events and percentages. For continuous variables, normally distributed data are presented as mean ± standard deviation (SD) and skewed data as median and interquartile range (IQR). The normality of distribution and variances were checked using histograms and the Kolmogorov-Smirnov test. The chi-square test, one-way ANOVA, or the Kruskal-Wallis test was used for comparison of baseline data and unadjusted outcomes. Bonferroni post-hoc test was performed when appropriate. No post-hoc test was conducted following the Kruskal-Wallis test. The Cochran-Armitage trend test (CATT) was used for all binary variables, where the two-level variable represents the response, and the CSN represents the explanatory variable. Logistic regressions were built to assess the prediction of the experience groups on in-hospital death, adjusting for potential confounding factors (old vs. new generation THV, transfemoral vs. non-transfemoral approach, valve-in-valve procedures, and EuroSCORE-II). Old-generation THV were those from the first line of devices commercially available in Brazil (Table S1). Other potential predictors of in-hospital death were checked but were not associated with the outcome in the present cohort: mean aortic valve gradient in baseline echo, bicuspid aortic valve, general anesthesia, percutaneous access, valve brand, balloon pre-dilation and balloon post-dilation. As EuroSCORE-II includes most variables from Table 1, these were not included in the model due to collinearity. P-values <0.05 were considered as statistically significant. Analyses were carried out using R [v.3.5.3].

**Table 1:** – Characteristics of the enrolled individuals

	1^st^ to 40^th^ case	41^st^ to 80^th^ case	81^st^ to 120^th^ case	Over the 121^st^ case	p value	CATT* p value
Sample size (n)	991	616	458	1129		
Age	80.9±7.3	80.9±7.5	81.7±7	79.9±9.3	<0.001	
Female	520 (52.5)	283 (45.9)	27 (49.6)	521 (46.1)	0.014	0.012
Diabetes	344 (34.8)	210 (34.1)	150 (32.8)	360 (32.1)	0.593	0.170
NYHA						
III	512 (51.8)	302 (49)	190 (41.8)	422 (47.3)		
IV	276 (27.9)	140 (22.7)	116 (25.5)	117 (13.1)		
Atrial fibrillation	22 (17.9)	13 (12)	20 (25)	42 (20.2)	0.133	0.255
Pacemaker	17 (8.3)	15 (7.9)	14 (8.9)	35 (9.3)	0.944	0.593
Previous MI	125 (13.5)	92 (16.3)	66 (15.8)	98 (9.6)	<0.001	0.007
Previous PCI	285 (29.1)	169 (27.9)	143 (31.3)	213 (22)	<0.001	0.002
Previous CABG	152 (5.5)	120 (19.5)	75 (16.4)	117 (12.6)	0.003	0.038
Previous Valve Surgery	6 (7.2)	4 (6.4)	2 (5)	14 (8.3)	0.668	0.958
Cerebrovascular Disease	144 (14.8)	82 (13.6)	52 (11.4)	87 (7.8)	<0.001	<0.001
Previous Stroke	71 (6.3)	36 (6)	28 (6.1)	61 (5.4)	0.335	0.089
Peripheral Vascular Disease	164 (16.9)	112 (18.5)	82 (17.9)	97 (8.7)	<0.001	<0.001
COPD	177 (18.3)	121 (20.0)	99 (21.7)	108 (9.6)	<0.001	<0.001
Creatinine, mg/dL	1.3±0.8	1.3±0.9	1.4±1.2	1.3±0.9	0.461	
Baseline Echo						
LVEF	59.1±13.9	58.9±14	59.1±12.9	58.3±13	0.005	
Mean Gradient	48±17.6	44.8±18	46.24±17.4	44±17.8	<0.001	
Bicuspid aortic valve	35 (5.2)	18 (3.7)	11 (3.1)	9 (3.0)	0.160	0.413
Surgical risk						
EuroSCORE-II	7.71 (8.00)	7.42 (6.64)	8.14 (8.25)	5.37 (5.75)	<0.001	

*Source: Bernardi, 2023. Values are n (%) or mean (±SD). CATT: Cochran-Armitage trend test; MI: myocardial infarction; PCI: percutaneous coronary intervention; CABG: coronary artery bypass graft; COPD: chronic obstructive pulmonary disease; LVEF: left ventricular ejection fraction.*

### Results

A total of 3,194 TAVR patients from 25 Brazilian centers were included. Ten cases were excluded due to lack of sufficient in-hospital information. The first case was performed in February 2008 and the last in February 2023 (Central Illustration). There were 111 missing cases for the variable transfemoral approach and 63 for prosthesis generation which were handled with multiple imputations.

### Baseline and procedural characteristics

Tables 1 and 2 summarize the baseline and procedural characteristics of the overall population and each experience group level. When comparing patients’ baseline data between the experience groups, the initial-, early- and intermediate- were quite balanced with similar mean ages and small differences in the rates of diabetes mellitus, coronary artery disease, peripheral arterial disease, and COPD, with a mean EuroSCORE II of 7.7±8, 7.4±6.6, 8.1±8.3, respectively. In the high-experience group, patients had fewer comorbidities and a significantly lower EuroSCORE II (5.4±5.7; p=0.012, p=0.019, and p<0.001 against the initial-, early- and intermediate experience, respectively).

**Table 2:** – Procedural characteristics of the enrolled patients

	1^st^ to 40^th^ case	41^st^ to 80^th^ case	81^st^ to 120^th^ case	Over the 121^st^ case	p value	CATT* p value
Sample size (n)	991	616	458	1129		
Year when half of the procedures were performed	April/2015	May/2016	January/2017	April/2019		
New-generation THV	363 (36.7)	303 (49.3)	292 (63.9)	66 (90)	<0.001	<0.001
Valve-in-valve	43 (4.4)	18 (3.1)	23 (5.4)	50 (4.9)	0.271	0.333
General anesthesia	744 (75.8)	409 (71.9)	324 (77)	401 (43.8)	<0.001	<0.001
Transfemoral approach	955 (96.8)	575 (93.8)	433 (95.1)	978 (96.4)		<0.001
Percutaneous Access	707 (71.6)	516 (84.2)	405 (89.2)	982 (96.9)	<0.001	<0.001
Balloon Predilation	406 (42.2)	257 (43.4)	184 (40.7)	368 (37.0)	0.042	0.012
Valve brand						
Sapien XT	188 (19)	134 (21.8)	81 (17.7)	55 (5.1)		
CoreValve	413 (41.8)	157 (25.6)	53 (11.6)	28 (2.6)		
Lotus	15 (1.5)	3 (0.5)	28 (6.1)	16 (1.5)		
Sapien S3	179 (18.1)	112 (18.2)	126 (27.6)	528 (49.2)		
Evolut R/PRO	30 (13.2)	167 (27.2)	141 (30.9)	251 (23.4)		
Braile	9 (0.9)	17 (2.8)	3 (0.7)	8 (0.7)		
Portico	11 (1.1)	0 (0)	0 (0)	8 (0.7)		
Acurate Neo/Neo2	41 (4.1)	16 (2.6)	17 (3.7)	146 (13.6)		
Myval	0 (0)	3 (0.5)	6 (1.3)	12 (1.1)		
Unreported	2 (0.2)	5 (0.8)	2 (0.4)	21 (2)		
Balloon Post-dilation	295 (30.8)	173 (31.1)	125 (29.8)	255 (28.1)	0.557	0.198
Valve Embolization	25 (2.7)	13 (2.7)	8 (2.2)	10 (1.1)	0.070	0.013
Need for 2nd Valve	27 (2.9)	19 (4)	9 (2.5)	11 (1.2)	0.010	0.008
Coronary artery occlusion	4 (0.5)	6 (1.4)	4 (1.2)	1 (0.1)	0.015	0.197
Annulus rupture	8 (0.9)	1 (0.2)	1 (0.3)	5 (0.5)	0.371	0.407
Tamponade	34 (3.7)	14 (3.1)	13 (3.9)	19 (2.3)	0.345	0.071
Conversion Open Surgery	34 (3.5)	12 (2.0)	10 (2.2)	11 (1.0)	0.001	<0.001

*Source: Bernardi, 2023. Values are n (%). CATT: Cochran-Armitage trend test.*

Most procedures were transfemoral (96%), with no significant difference between groups. However, as experience grew, a greater proportion of cases were treated by a percutaneous approach and without general anesthesia. Valve-in-valve accounted for only 4.4% of the procedures, with similar rates according to experience levels. The use of newer generation THV steadily increased as the centers’ experience grew, from 36.7% to 90% in the initial- and high-experience, respectively. Valve embolization, the need for a second valve, coronary obstruction, and conversion to open surgery were all less common at the high-experience stratum (p<0.05 for all).

### Learning curve and outcomes

Figure 1 illustrates the LC of TAVR with a spline regression for in-hospital death adjusted to log-transformed EuroSCORE II and the use of new-generation THV, showing that it was necessary to treat 40 cases until a first drop in the adjusted probability of mortality. A change in slope was observed at CS#118, signaling a leveling off for in-hospital mortality after this experience level. The end of the LC was determined to be at CS#303 based on the smallest upper bound of the 95% confidence interval of the logistic regression test that was below the significance level threshold.

When comparing the groups of experience, we observed a continuous drop in the unadjusted in-hospital mortality from the initial- (8.7%), early- (8%), intermediate- (6.1%), and high-experience (4.0%) (P< 0.001) (Figure 2). There was also a significant difference in the incidence of major vascular complications, major or life-threatening bleeding, and stroke. After adjusting for confounders and having the initial-experience as control, only the high-experience group was associated with a significant reduction of in-hospital mortality (OR 0.52, p=0.002). Transfemoral approach (OR 0.51, p=0.014) and the use of new-generation THV were also predictive of reduced hospital mortality (OR 0.69, p=0.029) along with lower EuroSCORE II (Figure 3).

**Figure 2:** – Univariate unadjusted post-transcatheter aortic valve replacement (TAVR) in-hospital outcomes according to case sequence number; A) Overall population; B) Centers with high-volume of TAVR procedures [≥40 procedures] before 2014; C) Centers with low-volume of TAVR procedures [<40 procedures] before 2014. CATT: Cochran-Armitage trend test.

**Figure 3:** – Forest plots for in-hospital death rate according to case sequence group at transcatheter aortic valve replacement (TAVR) centers with a greater volume (>40 cases) and centers with a lower volume (<40 cases) of patients treated before 2014.

### Centers with initial experience before and after 2014

Eight of the 25 centers concluded their initial experience (first 40 TAVR cases) before 2014, accounting for a total of 1,916 patients with a median number of procedures per center of 222 (IQR 163 to 282). The remaining 17 centers completed their initial experience after 2014, accounting for 1,278 patients with a median number of procedures per center of 56 (IQR 34.5 to 115). Supplemental tables S1 and S2 describe baseline and procedural characteristics of the groups (by level of experience) by case sequences in the centers with initial experience before and after 2014. Overall, patients had similar risk scores as determined by EuroSCORE II (mean of 7.0±7.5 and 6.9±6.8 for initial experience before and after 2014, respectively). In centers with initial experience after 2014, new-generation THVs were implanted significantly more often (70.3% vs. 55.4%, p<0.001).

As shown in Figure 4, the LC of the two cohorts differed in pattern. Among centers with initial experience concluded before 2014, a typical LC pattern was observed with an early initial drop in mortality occurring after the first 10 cases. A change in slope was observed at CS#81, signaling a leveling off for mortality after this experience level. Meanwhile, among centers with initial experience concluded after 2014 (Figure 5), we observed an initial lower mortality, but the curve remained steady until approximately case #100. Following that, the curve dropped, albeit with a steady widening of the confidence interval due to the lower number of centers with more than 100 cases in this cohort.

**Figure 4:** – Spline regressions for in-hospital death (adjusted to log-transformed EuroSCORE-II and the utilization of new generation THV) to determine the learning curve of centers with initial experience completed before 2014.

**Figure 5:** – Spline regressions for in-hospital death (adjusted to log-transformed EuroSCORE-II and the utilization of new generation transcatheter heart valve) to determine the learning curve of centers with initial experience completed after 2014.

In the cohort of centers that adopted TAVR before 2014, intermediate- and high-experience had an odds ratio of 0.47 (p=0.027) and 0.44 (p=0.003), respectively, for in-hospital mortality in comparison to initial-experience after adjusting for EuroSCORE II. We did not find a significant relationship between acquired experience and hospital mortality among late adopters (after 2014) (Figure 2). Figure 2 shows the unadjusted event rates of the two cohorts according to the different experience levels.

## Discussion

In this large nationwide TAVR registry in Brazil, we assessed the LC by analyzing the impact of the accumulated experience of the centers on in-hospital mortality, and the main results are as follows: 1) a total of 25 centers and 3,194 patients were included; an accumulated experience was associated with a reduction in in-hospital mortality, with the LC showing a first drop in mortality from case #40 until leveling off in case #118; 2) high-experience, determined by an accumulated experience of more than 120 cases, was an independent predictor of lower in-hospital mortality, being also associated with lower unadjusted rates of complications such as clinically relevant bleeding, major vascular complications, and stroke; 3) there were two distinct patterns of LC in centers that had their initial experience before and after 2014, suggesting a possible attenuation of in-hospital mortality by the accumulated experience of centers that began their TAVR programs later; 4) besides EuroSCORE II, transfemoral approach and the use of new-generation THV were other independent variables associated with lower in-hospital mortality.

The knowledge of the LC in complex procedures such as TAVR is crucial for planning processes that aim to continuously enhance clinical practices and optimize the allocation of future resources. Although previous studies have evaluated the LC of TAVR,^1-3,8-10^ our study is the first multicenter study conducted in a South American country, whose reality is vastly different from that of high-income countries. In Brazil and other developing nations, population access to TAVR has been significantly restricted,^4,5^ largely due to economic constraints and limited access to the procedure in the public health system.^11,12^ For instance, by 2017, less than 10 TAVR procedures were performed per one million inhabitants in Brazil, against more than 100-150 in countries like the USA, France, and Germany.^5^ This regional variation in TAVR accessibility and volume of procedures may be a factor in determining the LC in a country. In a continental nation such as Brazil, an analysis incorporating institutions from various regions of the country is of utmost importance.

Our findings are consistent with international multicenter studies that demonstrate a decline in the incidence of early adverse events as institutions gained experience.^1-3,8^ Importantly, we have observed a decrease in mortality from the case #40 on, similar to Russo et al.^1^ who also found an improvement from case #38. However, these findings contrast with the study of Wassef et al.^2^ who found an improvement beginning with case #75; both were large multicenter studies, mainly driven by institutions from high-income countries. This improvement in mortality after procedure #120 in our study, as well as beyond procedures #170 and #150 in the studies by Russo et al.^1^ and Wassef et al.,^2^ respectively. Despite distinct realities, these findings demonstrate that the overall LC in Brazil was somehow comparable to that from higher-income countries with much broader access to TAVR, reflecting that the procedure technique has an intrinsic LC related to the operator’s proficiency.

Nevertheless, it is essential to recognize that TAVR has evolved since its inception. In the past 15 years, numerous device iterations and refined techniques including less invasive approaches have emerged. Notably, a study that assessed the development of TAVR practices in Latin American centers, the majority of them from Brazil, revealed a significant change in practice between 2015 and 2020, with greater incorporation of minimalist procedures and universal adoption of the most recent versions of THVs.^4^ To determine whether these temporal changes in TAVR practices affected the LC, we have performed a sub-analysis, dividing centers with initial experience (first 40 cases performed before 2014) from those with initial experience completed after 2014, the year in which the first new-generation THV became commercially available in Brazil. Interestingly, the LC phenomenon, with an initial reduction of in-hospital mortality, was practically absent among late TAVR adopters, unlike the early adopters, where a clear initial LC was seen, with a reduction in the probability of death already occurring after the first ten cases. Likewise, Russo et al.,^1^ analyzing data from the North American TVT registry, found no evidence of a LC in centers where initial experience occurred after 2015 with the latest balloon-expandable prosthesis Sapien S3 (Edwards Lifesciences, Irvine, CA). These centers did not exhibit a significant improvement in clinical outcomes with increased experience, and their initial clinical outcomes were already comparable to those of centers with greater experience. According to the authors, this result was not surprising, as device improvements, intensive proctoring programs, and knowledge dissemination may result in more rapid adoption of TAVR techniques among newer and lower-volume centers.^1^

Many factors in the current practice may have contributed to this better start by new TAVR centers, such as: 1) increased general knowledge of the entire scientific community and an impressive accumulation of scientific evidence over the course of TAVR´s journey;^13^ 2) the increasing experience in the use of more modern, user-friendly, reliable and safer THVs, which led to enhanced clinical outcomes;^14-16^ 3) the intense work of the scientific community, in collaboration with the industry, to enhance prosthesis implant techniques, combined with extensive knowledge dissemination efforts;^17^ 4) improvement in patient selection with greater inclusion of lower surgical-risk patients, as well as refinement of imaging techniques for better procedural planning;^17^ 5) extensive proctoring programs for the TAVR centers provided by the THV companies.

However, even though we did not find a significant link between reducing in-hospital deaths and the experience level of centers that started TAVR later, there seems to be an improvement trend, especially after the first 120 cases. It is important to note that only four out of 17 institutions fell into the high-experience group in our study, which led to wide confidence intervals due to the smaller number of patients. This means we cannot rule out the possibility of a type II error. In a typical LC, we would expect better results early on. But in this case, we need to consider the possibility of a LC that progressed slowly in the beginning, followed by a gradual but smaller improvement, until full proficiency achieved by the centers that completed their initial TAVR experience after 2014. Even though these centers began with a lower in-hospital mortality rate (7.6% vs. 10.9% for the centers completing the initial experience before 2014), there was minimal or no improvement by the 120th case. Despite dealing with patients at moderate to high-risk (mean EuroSCORE II of nearly 8.0), in-hospital mortality rate was notably higher than that reported in other international studies. For instance, in a study from the TVT registry, institutions that began working with the Sapien S3 balloon-expandable prosthesis after 2015 consistently maintained a low hospital mortality rate of about 4%, even when dealing with intermediate to high-risk patients (mean STS score of 7.3%).^1^ Therefore, the lack of immediate improvement as the case numbers increased in our analysis suggests that these newer TAVR institutions may have experienced an initial slow learning process. Additionally, the relatively high occurrence of vascular complications in our cohort, which is often seen as a marker of TAVR expertise, further supports this hypothesis.

A question remains as to why the learning process of these centers was delayed in terms of reduced in-hospital mortality. A possible explanation may be the limited volume of TAVR performed at these institutions. Before case #120, the median annual number of procedures was only 6.4 (IQR 5 to 11) compared to 15 (IQR 12.3 to 17.1) for the cohort of centers that had their initial experience before 2014. Previous studies have consistently demonstrated a significant association between the volume of procedures and enhancement of outcomes, including short-term mortality.^2,18^ Nevertheless, our study was not designed to assess the impact of procedure volume of the centers, as this analysis would be complex to interpret considering that many TAVR hospitals have initiated their programs during the analyzed period, and many operated with limited case volumes (<20 TAVR/year), posing a statistical challenge for an analysis of hospital-based outcomes. Given the imminent inclusion of the procedure in Brazil’s public health system, such information ought to be duly considered in future studies for further advancement of the field in the country. Therefore, it is of utmost importance to continue with the detailed analysis of data from the Brazilian registry, as well as from other lower-income countries. This in-depth investigation is essential to examine the various factors associated with the LC in the specific context of these countries, thereby ensuring better outcomes and significant advancements in the treatment of TAVR in patients with aortic stenosis.

### Study limitations

This is an observational study with data from a real-world registry with site-reported outcomes and remote monitoring but no central adjudication. Even though outcomes standardized by the VARC-2 criteria were utilized, there could be inconsistencies in reporting endpoints. That was the reason why we chose all-cause in-hospital mortality for the primary analysis, to mitigate potential assessment bias. Moreover, we did not evaluate the impact of the LC on mid- and late-term outcomes as we assessed only in-hospital events. There was no assessment of the LC for individual operators at each center either. Also, although we adjusted the primary endpoint for EuroSCORE II to account for the increasing number of lower-risk patients being treated over time, it’s still important to consider the potential impact of unmeasured variables that may act as confounding factors. Regarding our sub-analysis, most late adopters were still close to their initial experience, which may have contributed to the lack of a significant decline in mortality and thus the experience effect may still be unnoticeable. Finally, TAVR continues evolve rapidly, with the development of more refined techniques and superior devices almost annually. Therefore, our findings may not fully represent the most up-to-date practice.

## Conclusion

Throughout the history of TAVR in Brazil, the accumulated experience of major institutions in the country has been associated with reduced in-hospital mortality indicating a true LC phenomenon. However, this relationship between initial experience and enhanced outcomes was more impactful during the early days of TAVR. For newer TAVR centers, despite better results in the beginning, there was a slower rate of clinical improvement as experience was gained. These findings contribute to the understanding of the LC in TAVR and provide insights for future research in this rapidly evolving field.
